# Analytical Formulation of Relationship Between Sensors and Euler Angle Errors for Arbitrary Stationary Alignment Based on Accelerometer and Magnetometer

**DOI:** 10.3390/s25082593

**Published:** 2025-04-19

**Authors:** Chang June Lee, Jung Keun Lee

**Affiliations:** 1 Department of Integrated Systems Engineering, Hankyong National University, Anseong 17579, Republic of Korea; cjlee@hknu.ac.kr; 2 School of ICT, Robotics & Mechanical Engineering, Hankyong National University, Anseong 17579, Republic of Korea

**Keywords:** analytical formulation, Euler angle error, inertial measurement unit, sensor error, stationary alignment

## Abstract

An attitude and heading reference system (AHRS) based on the inertial measurement unit is crucial for various applications. In an AHRS, stationary alignments are performed to determine the initial orientation of the sensor frame with respect to the navigation frame. However, the stationary alignment accuracy is affected by sensor error factors. Therefore, several studies have attempted to analyze and minimize the effects of these errors. However, there have been no studies describing and analyzing the Euler angle errors for various sensor orientations. This paper presents the analytical formulation of the relationship between the sensor and the Euler angle errors based on accelerometer and magnetometer signals, regardless of alignment between the sensor and the navigation frames. We selected three-axis attitude determination (TRIAD) as the stationary alignment method and considered the scale, installation, and the offset errors, including noise and constant bias, as sensor error factors. The presented formulation describes the relationship between the sensor error factors and the Euler angle errors as a linear equation. To analyze the Euler angle errors, we performed both sensor-aligned and sensor-misaligned simulations in which the Euler angles were 0° and arbitrary, respectively. The results showed that the presented error formulation could describe the total Euler angle errors and the partial errors induced by each sensor error factor for both the sensor-aligned conditions and the arbitrary Euler angle configurations. Thus, the effects of each sensor error factor on the Euler angle errors can be analytically investigated using the presented formulations for random alignment.

## 1. Introduction

Attitude and heading reference systems (AHRSs) play important roles in various applications, such as robotics, unmanned vehicles, spacecraft, and wearable systems for human motion tracking [1,2,3,4,5,6,7]. For example, to control aerial or underwater unmanned vehicles, the 3D orientation of a vehicle must be provided in real time. Therefore, most unmanned vehicles are equipped with an AHRS module [1,2,3,4]. In addition, an inertial motion capture system used to kinematically analyze human motion is equipped with multiple AHRS modules to measure the 3D orientation of each body segment [5,6,7]. Furthermore, the AHRS is essential for strapdown inertial navigation systems (SINSs) to estimate velocity and position [8,9,10].

In an AHRS, a microelectromechanical system (MEMS)-based inertial measurement unit (IMU) is the core element that provides 3D orientation, including the attitude and heading of the sensor module. An IMU generally comprises a three-axis accelerometer and a three-axis gyroscope, and it is often combined with a three-axis magnetometer, which is a 3D electronic compass. The gyroscope provides a rotational angular velocity signal that can be used for strapdown integration [11,12]. Accelerometers and magnetometers provide gravitational acceleration and geomagnetic field signals, respectively, which can be used as vertical and horizontal reference vectors [13,14,15,16]. However, each of the three IMU sensors has several inaccurate factors in 3D orientation estimation in AHRS applications. Thus, many studies have developed filtering or machine learning algorithms that combine the signals from three sensors to improve orientation estimation during dynamic movements and magnetic disturbances [17,18,19,20,21,22]. After determining the orientation using the AHRS, the SINS computes linear acceleration from the accelerometer signals and the sensor orientation to subsequently estimate the velocity and position [8,9,10].

Both the AHRS and the SINS require a stationary alignment procedure to determine the initial orientation of the sensor frame with respect to the navigation frame. The basic concept of stationary alignment involves determining sensor orientation based on the reference vectors observed in the sensor frame and those known in the navigation frame [23,24]. Conventionally, stationary alignment is performed based on gravitational acceleration and earth rate vectors using accelerometer and gyroscope signals [25,26,27,28,29]. However, this approach requires an expensive gyroscope that can measure Earth’s rate with high reliability [30]. Therefore, in many studies, a magnetometer was used as an alternative to the gyroscope as a magnetometer can exploit the observed/known vectors of Earth’s magnetic field for stationary alignment [13,14,15,16].

Alignment accuracy depends on the performance of the alignment algorithm, dynamic and magnetic disturbances, and sensor error factors. Sensor errors, such as scale errors, installation errors (alignment and orthogonality), and offset errors (noise and bias), can lead to inaccuracies in sensor signal acquisition [31,32]. Therefore, research has been conducted to analyze and compensate for the effects of IMU error factors. Several studies developed sensor calibration methods based on sensor error modeling and presented calibration procedures without external equipment or with equipment such as motor systems [33,34,35,36,37]. For example, Zhao et al. [37] developed a calibration method that compensates for installation errors by decomposing them into nonorthogonal and misalignment errors and calibrating the sensor using a three-axis turntable system.

Whereas the above studies focused only on calibrating sensor errors, some studies also analyzed the effects of sensor errors on alignment or navigation accuracy [38,39,40]. For example, Zheng et al. [38] analyzed the effects of three deterministic errors (bias, scale error, and installation error) on the accuracy of attitude, velocity, and position in accelerometer- and gyroscope-based navigation systems. Regarding the error analysis of stationary alignment, Silva et al. [39] presented error analysis formulations of normality, orthogonality, the alignment errors of the direction cosine matrix, and the Euler angle error for five stationary alignment formulations based on accelerometer and gyroscope signals. Their follow-up study [40] expanded the error analysis formulation to include four stationary alignment methods based on accelerometer and magnetometer signals. Some studies [39,40] analytically described the relationship between the sensor error and orientation error, but these studies assumed that the sensor frame was perfectly aligned with the navigation frame. In addition, Euler angle error analysis for accelerometer- and magnetometer-based stationary alignments was not addressed in [40]. Because Euler angles provide an intuitive representation that is primarily used to analyze 3D orientation, it would be helpful to analyze the Euler angle errors owing to accelerometer and magnetometer errors.

This study developed an analytical formulation of the relationship between the sensor errors and the Euler angle errors for stationary alignment based on an accelerometer and magnetometer, regardless of the alignment between the sensor and the navigation frames. In the presented formulation, the relationship between the sensor error factors and the Euler angle errors was described using a linear equation. In this study, we compared and analyzed the analytical formulations using a numerical approach. Both a sensor-aligned situation, in which the sensor frame was aligned with the navigation frame (i.e., the roll, pitch, and yaw angles were all 0°), and a sensor-misaligned situation, in which the sensor frame deviated from the navigation frame (i.e., the Euler angles were arbitrary), were considered.

## 2. Materials and Methods

### 2.1. Three-Axis Attitude Determination (TRIAD)

**Figure 1:**
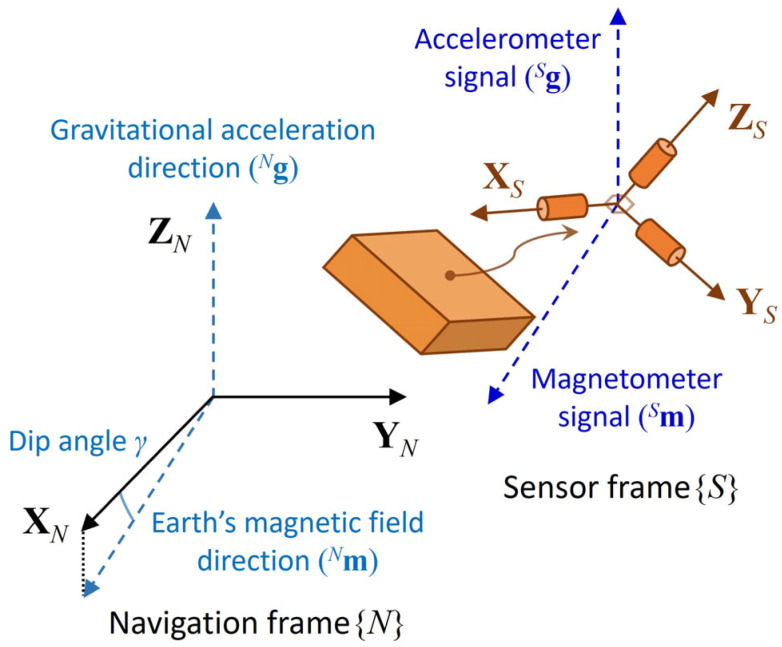
Navigation and sensor frames.

This study employed a three-axis attitude determination (TRIAD) method for stationary alignment using accelerometer and magnetometer signals [23,24]. The purpose of TRIAD is to determine a direction cosine matrix representing the orientation of the sensor frame {*S*} with respect to the navigation frame {*N*}, where the X-axis is magnetically north and the Z-axis is vertical (Figure 1).

The direction cosine matrix comprising the three axes (X, Y, and Z) unit vectors is described as follows [41]:

(1) RSN=r11r12r13r21r22r23r31r32r33=XSNYSNZSN=XNTSYNTSZNTS,

where the left superscripts *N* and *S* indicate that the vector is observed at {*N*} and {*S*}, respectively, and the right superscript *T* indicates the transposition of the vector or matrix. According to the Z-Y-X Euler angles, the direction cosine matrix can be rewritten in terms of roll (*ϕ*), pitch (*θ*), and yaw (*ψ*) angles as follows [41]:

(2) RSN=cθcψ−cϕsψ+sϕsθcψsϕsψ+cϕsθcψcθsψcϕcψ+sϕsθsψ−sϕcψ+cϕsθsψ−sθsϕcθcϕcθ,

where *cϕ* and *sϕ* indicate the cosine and sine of angle *ϕ*, respectively.

Under undisturbed conditions, where the sensor acceleration and magnetic disturbance are negligible, the ideal signals of the accelerometer (*A*) and the magnetometer (*M*) are described as follows:

(3a) yA=axayazT=gS=RTSN gN,

(3b) yM=mxmymzT=mS=RTSN mN,

where **g** is the acceleration vector owing to the reaction to gravity, and **m** is Earth’s magnetic field vector. These two vectors, known in the navigation frame, are described as follows:

(4a) gN=g⋅001T,

(4b) mN=m⋅cγ0sγT,

where *γ* is the magnetic dip angle. Based on Equations (2)–(4), the ideal signals of the accelerometer and the magnetometer can be rewritten as follows:

(5a) $$y_{A}=g\cdot {\left[\begin{array}{ccc} -s\theta & s\phi c\theta & c\phi c\theta \end{array}\right]}^{T},$$

(5b) $$y_{M}=m\cdot \left[\begin{array}{c} c\theta c\psi c\gamma -s\theta s\gamma \\ \left(-c\phi s\psi +s\phi s\theta c\psi \right)c\gamma +s\phi c\theta s\gamma \\ \left(s\phi s\psi +c\phi s\theta c\psi \right)c\gamma +c\phi c\theta s\gamma \end{array}\right].$$

TRIAD uses two matrices comprising three reference vectors that are known in the navigation frame and observed in the sensor frame [23,24]. Originally, all the three reference vectors in each matrix were unitary vectors that were orthogonal to each other. However, the analytical formulation proposed in this study intentionally allows for slight violations of the unitary and orthogonal constraints on vector observations. This choice was made to achieve a balance between mathematical simplicity and practical applicability. Thus, in this study, the following two matrices were used without normalization to the unitary vectors or the application of orthogonalization [40]:

(6a) MN=gNmNgN×mN,

(6b) $$M_{S}=\left[\begin{array}{ccc} y_{A} & y_{M} & y_{A}\times y_{M} \end{array}\right].$$

Using these two matrices, the direction cosine matrix R^SN is determined as follows:

(7) R^SN=MN−1TMST.

To obtain the inverse matrix of **M***_N_*, the determinant and the adjoint matrix were determined as follows:

(8a) $$\det M_{N}=m^{2}g^{2}c\gamma^{2}.$$

(8b) $$adjM_{N}=\left[\begin{array}{ccc} -g\cdot m^{2}\cdot c\gamma s\gamma & 0 & g\cdot m^{2}\cdot c\gamma^{2}\\ g^{2}\cdot m\cdot c\gamma & 0 & 0\\ 0 & g\cdot m\cdot c\gamma & 0 \end{array}\right].$$

The matrix R^SN determined by Equation (7) can be expanded as follows:

(9) R^SN=−tγ⋅axg+mxm⋅cγ−tγ⋅ayg+mym⋅cγ−tγ⋅azg+mzm⋅cγaymz−azmyg⋅m⋅cγazmx−axmzg⋅m⋅cγaxmy−aymxg⋅m⋅cγaxgaygazg.

Here, *tγ* is the tangent of angle *γ*. Based on the Z-Y-X Euler angles, the tangents of the roll, pitch, and yaw angles were calculated as follows:

(10a) $$\bar{\phi }=\tan\phi =\frac{r_{32}}{r_{33}}=\frac{a_{y}}{g}\cdot {\left(\frac{a_{z}}{g}\right)}^{-1}=\frac{a_{y}}{a_{z}},$$

(10b) $$\bar{\theta }=\tan\theta =\frac{-r_{31}}{\sqrt{r_{11}^{2}+r_{21}^{2}}}=\frac{-k_{1}a_{x}}{\sqrt{{\left(k_{2}a_{x}+k_{3}m_{x}\right)}^{2}+k_{1}^{2}k_{3}^{2}{\left(a_{y}m_{z}-a_{z}m_{y}\right)}^{2}}},$$

(10c) $$\bar{\psi }=\tan\psi =\frac{r_{21}}{r_{11}}=\frac{k_{1}k_{3}\left(a_{y}m_{z}-a_{z}m_{y}\right)}{k_{2}a_{x}+k_{3}m_{x}}.$$

Here, *k*_1_, *k*_2_, and *k*_3_ are defined as follows:

(11) $$k_{1}=\frac{1}{g},\;\;k_{2}=-\frac{t\gamma }{g},\;\;k_{3}=\frac{1}{m\cdot c\gamma }.$$

Finally, the Euler angles were obtained by taking the arctangent of Equations (10a)–(10c).

### 2.2. Sensor Error Model

**Figure 2:**
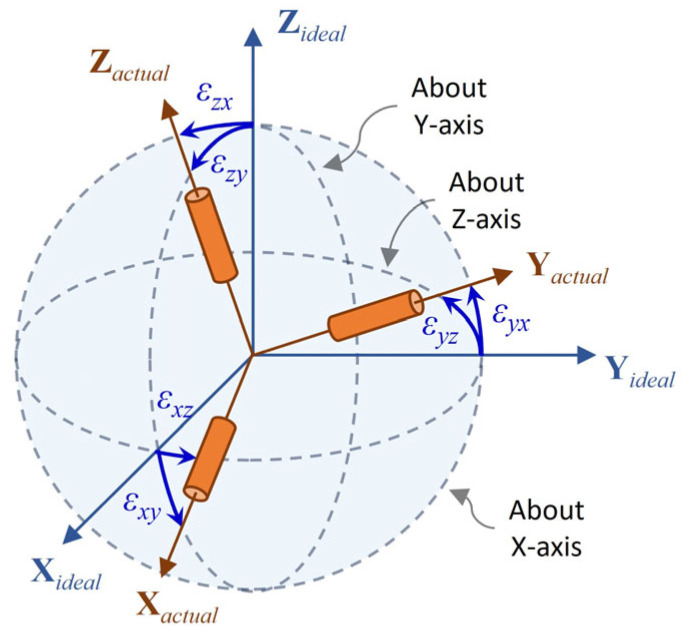
Illustration of sensor installation error.

The accelerometer and magnetometer signals considering the error factors were modeled as follows [38]:

(12a) $${\hat{y}}_{A}=\left(I+E_{A}\right)y_{A}+o_{A},$$

(12b) $${\hat{y}}_{M}=\left(I+E_{M}\right)y_{M}+o_{M},$$

where **E** is an error matrix that includes scale and installation errors, and **o** is an offset error vector that includes noise **n** and constant bias **b** (i.e., **o** = **n** + **b**). The sensor noise was modeled as a combination of white Gaussian noise and flicker noise; characterized by noise density and bias instability, respectively; and generated using the discrete error model proposed in [32]. According to [37,38], the error matrix **E** can be simplified under the assumption that the installation error angles are extremely small:

(13) $$E=\left[\begin{array}{ccc} k_{x} & -\varepsilon_{yz} & \varepsilon_{zy}\\ \varepsilon_{xz} & k_{y} & -\varepsilon_{zx}\\ -\varepsilon_{xy} & \varepsilon_{yx} & k_{z} \end{array}\right],$$

where *k_x_*, *k_y_*, and *k_z_* are scale factors for each axis, and *ε_yz_*, *ε_zy_*, *ε_xz_*, *ε_zx_*, *ε_xy_*, and *ε_yx_* are installation error angles (in radians) between the ideal and actual sensor axes (Figure 2).

The errors of the accelerometer and magnetometer signals modeled as in Equation (12) are calculated as follows:

(14a) $$\delta {\hat{y}}_{A}={\left[\begin{array}{ccc} \delta {\hat{a}}_{x} & \delta {\hat{a}}_{y} & \delta {\hat{a}}_{z} \end{array}\right]}^{T}={\hat{y}}_{A}-y_{A},$$

(14b) $$\delta {\hat{y}}_{M}={\left[\begin{array}{ccc} \delta {\hat{m}}_{x} & \delta {\hat{m}}_{y} & \delta {\hat{m}}_{z} \end{array}\right]}^{T}={\hat{y}}_{M}-y_{M}.$$

Based on Equation (12), the sensor errors can be expanded as follows:

(15a) $$\delta {\hat{y}}_{A}=\left[\begin{array}{c} k_{A,x}a_{x}-\varepsilon_{A,yz}a_{y}+\varepsilon_{A,zy}a_{z}+o_{A,x}\\ \varepsilon_{A,xz}a_{x}+k_{A,y}a_{y}-\varepsilon_{A,zx}a_{z}+o_{A,y}\\ -\varepsilon_{A,xy}a_{x}+\varepsilon_{A,yx}a_{y}+k_{A,z}a_{z}+o_{A,z} \end{array}\right],$$

(15b) $$\delta {\hat{y}}_{M}=\left[\begin{array}{c} k_{M,x}m_{x}-\varepsilon_{M,yz}m_{y}+\varepsilon_{M,zy}m_{z}+o_{M,x}\\ \varepsilon_{M,xz}m_{x}+k_{M,y}m_{y}-\varepsilon_{M,zx}m_{z}+o_{M,y}\\ -\varepsilon_{M,xy}m_{x}+\varepsilon_{M,yx}m_{y}+k_{M,z}m_{z}+o_{M,z} \end{array}\right].$$

From Equation (15), the following matrix equation is derived with respect to the sensor error factors:

(16) $$\left[\begin{array}{c} \delta y_{A}\\ \delta y_{M} \end{array}\right]=\left[\begin{array}{cc} C_{A} & O_{3\times 12}\\ O_{3\times 12} & C_{M} \end{array}\right]\left[\begin{array}{c} e_{A}\\ e_{M} \end{array}\right],$$

where **e***_A_* and **e***_M_* are vectors representing the scale, installation, and offset errors of the accelerometer and magnetometer signals, respectively, and are determined as follows:

(17a) $$e_{A}={\left[\begin{array}{ccc} k_{A}^{T} & \varepsilon_{A}^{T} & o_{A}^{T} \end{array}\right]}^{T},$$

(17b) $$e_{M}={\left[\begin{array}{ccc} k_{M}^{T} & \varepsilon_{M}^{T} & o_{M}^{T} \end{array}\right]}^{T},$$

where **k** is a vector including three scale factors, and **ε** is a vector that includes six installation error angles as follows:

(18) $$\varepsilon ={\left[\begin{array}{cccccc} \varepsilon_{yz} & \varepsilon_{zy} & \varepsilon_{xz} & \varepsilon_{zx} & \varepsilon_{xy} & \varepsilon_{yx} \end{array}\right]}^{T},$$

Additionally, **C***_A_* and **C***_M_* are conversion matrices from error factors to sensor signal errors and are determined as follows:

(19a) $$C_{A}=\left[\begin{array}{cccccccccccc} a_{x} & 0 & 0 & -a_{y} & a_{z} & 0 & 0 & 0 & 0 & 1 & 0 & 0\\ 0 & a_{y} & 0 & 0 & 0 & a_{x} & -a_{z} & 0 & 0 & 0 & 1 & 0\\ 0 & 0 & a_{z} & 0 & 0 & 0 & 0 & -a_{x} & a_{y} & 0 & 0 & 1 \end{array}\right],$$

(19b) $$C_{M}=\left[\begin{array}{cccccccccccc} m_{x} & 0 & 0 & -m_{y} & m_{z} & 0 & 0 & 0 & 0 & 1 & 0 & 0\\ 0 & m_{y} & 0 & 0 & 0 & m_{x} & -m_{z} & 0 & 0 & 0 & 1 & 0\\ 0 & 0 & m_{z} & 0 & 0 & 0 & 0 & -m_{x} & m_{y} & 0 & 0 & 1 \end{array}\right].$$

### 2.3. Euler Angle Error Model

In this study, the Euler angle errors were derived using analytical and numerical approaches. In the analytical approach, a linear equation for the Euler angle errors is derived with respect to the errors in the accelerometer and magnetometer signals. Based on Equation (10), the tangent errors of the Euler angle can be determined using the errors of the accelerometer and magnetometer signals as follows:

(20) $$\left[\begin{array}{c} \delta \bar{\phi }\\ \delta \bar{\theta }\\ \delta \bar{\psi } \end{array}\right]=\left[\begin{array}{cc} J_{A} & J_{M} \end{array}\right]\left[\begin{array}{c} \delta {\hat{y}}_{A}\\ \delta {\hat{y}}_{M} \end{array}\right],$$

where **J***_A_* and **J***_M_* are Jacobian matrices representing the partial derivatives of the Euler angle errors with respect to the accelerometer and magnetometer errors, respectively, which are defined as follows:

(21a) $$J_{A}=\left[\begin{array}{ccc} J_{A(1,1)} & J_{A(1,2)} & J_{A(1,3)}\\ J_{A(2,1)} & J_{A(2,2)} & J_{A(2,3)}\\ J_{A(3,1)} & J_{A(3,2)} & J_{A(3,3)} \end{array}\right]=\left[\begin{array}{ccc} \frac{\partial \bar{\phi }}{\partial a_{x}} & \frac{\partial \bar{\phi }}{\partial a_{y}} & \frac{\partial \bar{\phi }}{\partial a_{z}}\\ \frac{\partial \bar{\theta }}{\partial a_{x}} & \frac{\partial \bar{\theta }}{\partial a_{y}} & \frac{\partial \bar{\theta }}{\partial a_{z}}\\ \frac{\partial \bar{\psi }}{\partial a_{x}} & \frac{\partial \bar{\psi }}{\partial a_{y}} & \frac{\partial \bar{\psi }}{\partial a_{z}} \end{array}\right],$$

(21b) $$J_{M}=\left[\begin{array}{ccc} J_{M(1,1)} & J_{M(1,2)} & J_{M(1,3)}\\ J_{M(2,1)} & J_{M(2,2)} & J_{M(2,3)}\\ J_{M(3,1)} & J_{M(3,2)} & J_{M(3,3)} \end{array}\right]=\left[\begin{array}{ccc} \frac{\partial \bar{\phi }}{\partial m_{x}} & \frac{\partial \bar{\phi }}{\partial m_{y}} & \frac{\partial \bar{\phi }}{\partial m_{z}}\\ \frac{\partial \bar{\theta }}{\partial m_{x}} & \frac{\partial \bar{\theta }}{\partial m_{y}} & \frac{\partial \bar{\theta }}{\partial m_{z}}\\ \frac{\partial \bar{\psi }}{\partial m_{x}} & \frac{\partial \bar{\psi }}{\partial m_{y}} & \frac{\partial \bar{\psi }}{\partial m_{z}} \end{array}\right].$$

Here, the elements of the Jacobian matrices are as follows:

(22) $$J_{A}=\left[\begin{array}{ccc} 0 & \frac{1}{a_{z}} & -\frac{a_{y}}{a_{z}^{2}}\\ \frac{-k_{1}K+k_{1}a_{x}\left(k_{2}^{2}a_{x}+k_{2}k_{3}m_{x}\right)}{K^{3/2}} & \frac{k_{1}^{3}k_{3}^{2}a_{x}\left(a_{y}m_{z}^{2}-a_{z}m_{y}m_{z}\right)}{K^{3/2}} & \frac{k_{1}^{3}k_{3}^{2}a_{x}\left(a_{z}m_{y}^{2}-a_{y}m_{y}m_{z}\right)}{K^{3/2}}\\ -\frac{k_{1}k_{2}k_{3}\left(a_{y}m_{z}-a_{z}m_{y}\right)}{{\left(k_{2}a_{x}+k_{3}m_{x}\right)}^{2}} & \frac{k_{1}k_{3}m_{z}}{\left(k_{2}a_{x}+k_{3}m_{x}\right)} & -\frac{k_{1}k_{3}m_{y}}{\left(k_{2}a_{x}+k_{3}m_{x}\right)} \end{array}\right],$$

(23) $$J_{M}=\left[\begin{array}{ccc} 0 & 0 & 0\\ \frac{k_{1}a_{x}\left(k_{3}^{2}m_{x}+k_{2}k_{3}a_{x}\right)}{K^{3/2}} & \frac{k_{1}^{3}k_{3}^{2}a_{x}\left(a_{z}^{2}m_{y}-a_{y}a_{z}m_{z}\right)}{K^{3/2}} & \frac{k_{1}^{3}k_{3}^{2}a_{x}\left(a_{y}^{2}m_{z}-a_{y}a_{z}m_{y}\right)}{K^{3/2}}\\ -\frac{k_{1}k_{3}^{2}\left(a_{y}m_{z}-a_{z}m_{y}\right)}{{\left(k_{2}a_{x}+k_{3}m_{x}\right)}^{2}} & -\frac{k_{1}k_{3}a_{z}}{\left(k_{2}a_{x}+k_{3}m_{x}\right)} & \frac{k_{1}k_{3}a_{y}}{\left(k_{2}a_{x}+k_{3}m_{x}\right)} \end{array}\right].$$

Here, *K* is defined as

(24) $$K={\left(k_{2}a_{x}+k_{3}m_{x}\right)}^{2}+k_{1}^{2}k_{3}^{2}{\left(a_{y}m_{z}-a_{z}m_{y}\right)}^{2}.$$

Euler angle errors are then obtained from their tangent errors as follows:

(25a) $$\delta \phi =\frac{\partial \phi }{\partial \bar{\phi }}\delta \bar{\phi }=\frac{1}{1+{\bar{\phi }}^{2}}\delta \bar{\phi },$$

(25b) $$\delta \theta =\frac{\partial \theta }{\partial \bar{\theta }}\delta \bar{\theta }=\frac{1}{1+{\bar{\theta }}^{2}}\delta \bar{\theta },$$

(25c) $$\delta \psi =\frac{\partial \psi }{\partial \bar{\psi }}\delta \bar{\psi }=\frac{1}{1+{\bar{\psi }}^{2}}\delta \bar{\psi }.$$

Using Equations (16), (20), and (26), the linear equation of the relationship between the sensor error factors and Euler angle errors can be derived as follows:

(26) $$\left[\begin{array}{c} \delta \phi \\ \delta \theta \\ \delta \psi \end{array}\right]=T\left[\begin{array}{c} e_{A}\\ e_{M} \end{array}\right],$$

where **T** is a linear transformation matrix from the sensor error factors to the Euler angle errors, which is described as follows:

(27) $$T=\left[\begin{array}{ccc} \frac{1}{1+{\bar{\phi }}^{2}} & 0 & 0\\ 0 & \frac{1}{1+{\bar{\theta }}^{2}} & 0\\ 0 & 0 & \frac{1}{1+{\bar{\psi }}^{2}} \end{array}\right]\left[\begin{array}{cc} J_{A} & J_{M} \end{array}\right]\left[\begin{array}{cc} C_{A} & O_{3\times 12}\\ O_{3\times 12} & C_{M} \end{array}\right],$$

**Figure 3:**
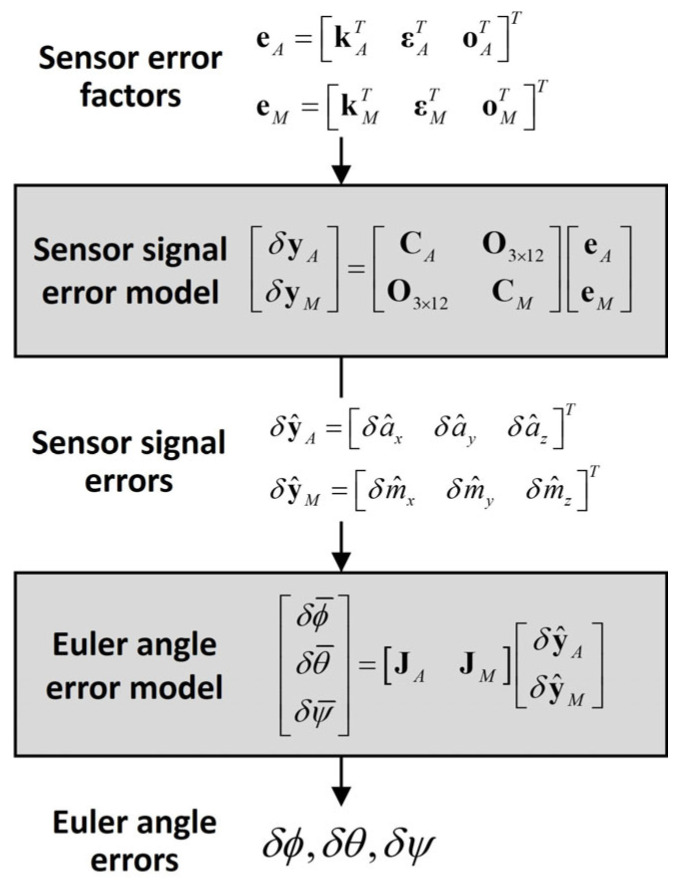
Flowchart of proposed error analysis method.

Here, the matrices **J** and **C** consist of the accelerometer and magnetometer signals, respectively, which are dependent on sensor orientation, that is, the Euler angles. Therefore, linear transformation matrix **T** can be considered a function of the Euler angles, i.e., **T** = *f*(*ϕ*, *θ*, *ψ*). Figure 3 shows a flowchart of the proposed error analysis.

Analytical error equations are not necessary for the numerical approach. First, the direction cosine matrix is estimated by directly substituting the sensor signal modeled in Equation (12) into Equation (6). Subsequently, the Euler angle error was determined using the Euler angle extracted from the estimated direction cosine matrix and the ground truth of the Euler angle.

## 3. Results

### 3.1. Simulation Test

In this study, we performed simulation tests to compare and analyze the Euler angle errors using analytical and numerical approaches. To generate two reference vectors *^N^***g** and *^N^***m** using Equation (4), the magnitude of gravitational acceleration (1 g) was set to 9.8 m/s^2^, the magnitude of the Earth’s magnetic field was set to 0.5 G, and the magnetic dip angle was set to −53° (Anseong, Korea).

**Figure 4:**
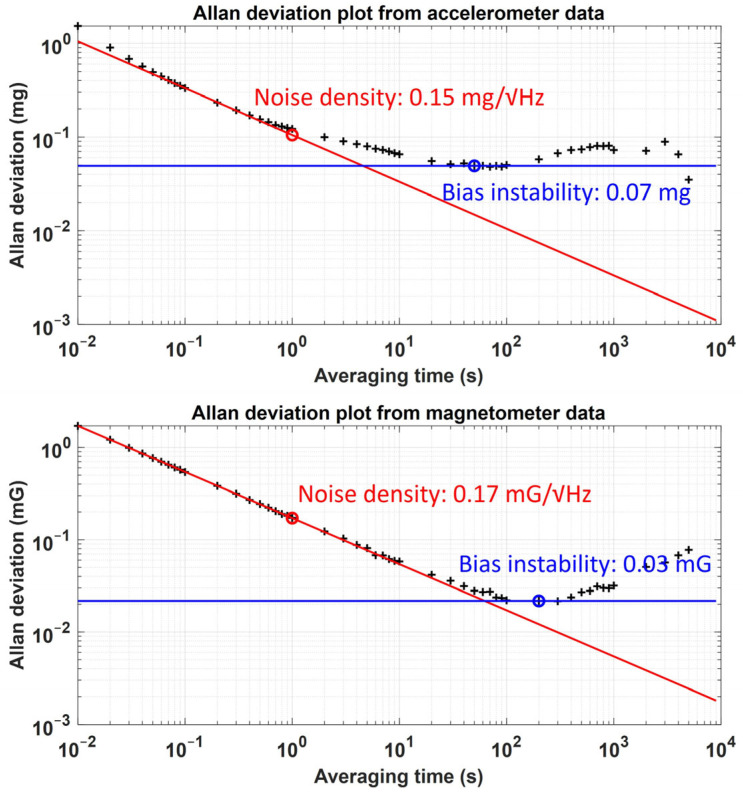
Allan deviation analysis results of accelerometer and magnetometer data.

We considered scale, installation, and offset errors, including noise and bias, as the sensor error factors. The installation error and noise parameters of the accelerometer and the magnetometer were selected based on the commercial IMU module MTw (Xsens Technologies, Enschede, The Netherlands). The sensor misalignment and orthogonality errors of MTw were described to be within 0.1° according to the user manual. Accordingly, the installation error angle was set as 0.1°. To obtain the noise densities and bias instabilities of the accelerometer and the magnetometer, we collected the MTw data under static and uniform magnetic conditions for 10,000 s at a sampling rate of 100 Hz. Subsequently, Allan deviation analysis was performed on the collected data. Figure 4 shows the Allan deviation results for accelerometer and magnetometer noise. According to the analysis results, the noise densities of the accelerometer and the magnetometer were set to 0.15 mg/√Hz and 0.17 mG/√Hz, respectively, and the bias instabilities of the two sensors were set to 0.07 mg and 0.03 mG, respectively.

We generated accelerometer and magnetometer noise using the discrete error model from [32], which is based on noise densities and bias instabilities. The scale factor of the two sensors was set to 0.1% (*k_A_*, *k_M_* = 0.001). We also compared the Euler angle errors caused by variations in the constant offset bias, considering that the initial sensor bias can differ each time the sensor is activated (i.e., turn-on bias stability) [32]. The accelerometer and magnetometer biases were applied from 0% to 0.1% (1 mg and 0.5 mG) at intervals of 0.01% of the magnitudes of gravitational acceleration and Earth’s magnetic field, respectively. Table 1 lists the selected values of the error factors, including the scale error, installation error, noise density, and constant bias of the accelerometer and the magnetometer.

In this study, we conducted sensor-aligned and sensor-misalignment simulations. For the sensor-aligned simulation, the sensor signals were generated under the condition that the sensor frame was aligned with the navigation frame. That is, the roll, pitch, and yaw angles were all 0°. The sensor-misaligned simulation was performed for 1000 trials, where an arbitrary Euler angle set was simulated for each trial. To generate an arbitrary sensor orientation for each trial, the standard deviation of the Euler angles between the trials was set to 20°. In both the simulations, ideal sensor signals from the accelerometer and the magnetometer were generated for 10 s at a sampling rate of 100 Hz. Subsequently, the Euler angle error results from the analytical and numerical approaches were compared and analyzed as the sensor error factors. All simulations were performed using MATLAB version 2023b.

### 3.2. Analysis of Results

First, in the sensor-aligned simulation results, the sensor signal and Euler angle errors were analyzed when the scale, installation, and zero-bias offset errors were applied. Table 2 shows the average and standard deviation of the accelerometer and magnetometer errors in the 10 s sensor-aligned simulation. Ideally, according to Equation (3), the accelerometer and magnetometer signals should coincide with the two reference vectors *^N^***g** and *^N^***m** because the direction cosine matrix is equal to the identity matrix in the sensor-aligned situation. However, the actual sensor signals suffer from signal distortion because of the sensor error factors (Equation (15)). When deterministic errors, such as scale and installation errors, and stochastic errors such as noise are applied, it is assumed that the deterministic errors primarily contribute to the average error, whereas stochastic errors influence the error deviations in terms of sensor signal errors. For example, when only the scale and installation errors were considered, the accelerometer errors were 1.7453, 1.7453, and 0.9970 mg for the X-, Y-, and Z-axes, respectively, whereas the magnetometer errors were 0.3969, 1.2221, and 0.9233 mG for the X-, Y-, and Z-axes, respectively. These values correspond closely to the average sensor signal errors listed in Table 2. Conversely, the standard deviations of the sensor signal errors closely aligned with the standard deviation of the respective sensor noise, which was 1.5 mg for the accelerometer and 1.7 mG for the magnetometer.

Table 3 lists the average errors and standard deviations of the Euler angle errors obtained using numerical and analytical approaches. The differences in average errors and standard deviations between the two approaches were within 0.001°. In terms of the average absolute differences, the differences in the roll, pitch, and yaw errors were 0.0001°, 0.0008°, and 0.0024°, respectively. Although the difference is larger for the yaw error than for the roll and pitch errors, considering the magnitude of the Euler angle errors, this difference seems to be negligible. Figure 4 shows the Euler angle errors resulting from the two approaches.

**Figure 5:**
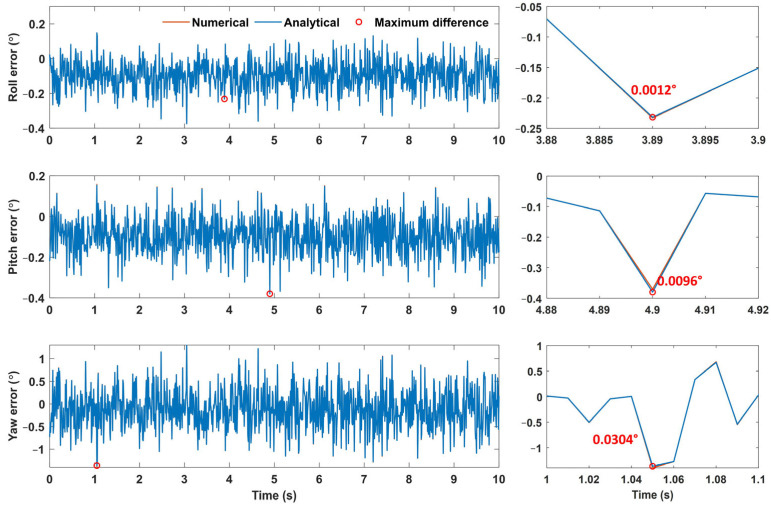
The Euler angle error results from the numerical and analytical approaches and maximum difference between their results in the sensor-aligned simulation (unit: °).

As shown in Figure 5, the results of the two approaches demonstrate excellent agreement for all the roll, pitch, and yaw errors, resulting in only the blue solid line (representing the analytical results) being visible. These results confirm that the linear analytical approach may produce Euler angle errors that are nearly identical to those obtained using the numerical approach. The maximum differences between the two approaches were found to be 0.0012°, 0.0096°, and 0.0304° for roll, pitch, and yaw, respectively.

**Figure 6:**
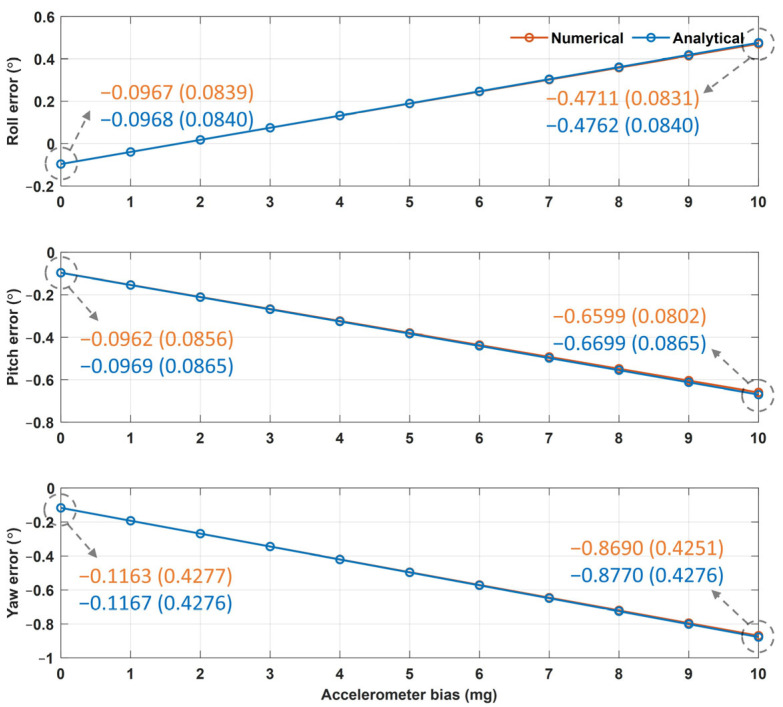
The Euler angle error results from the numerical and analytical approaches according to the accelerometer bias (unit: °).

**Figure 7:**
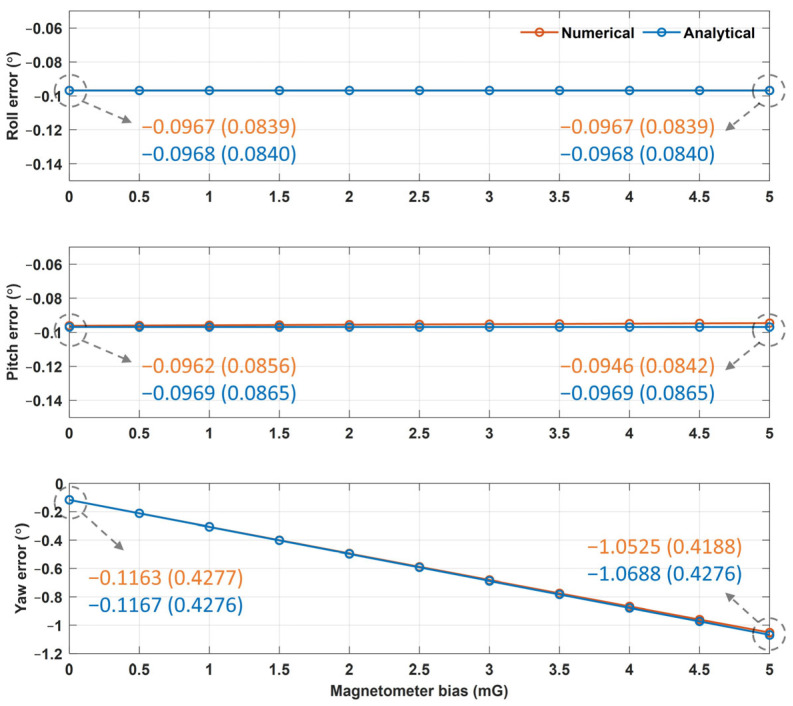
The Euler angle error results from the numerical and analytical approaches according to the magnetometer bias (unit: °).

Figure 6 and Figure 7 show the averages and standard deviations of the Euler angle errors from the numerical and analytical approaches based on the values of the accelerometer and magnetometer biases, respectively. The difference between the numerical and analytical approaches was found to be less than 0.001°. As shown in Figure 6, as the accelerometer bias increased from 0 to 1 mg, the pitch and yaw errors increased by approximately 0.057° and 0.076°, respectively, whereas the roll errors decreased by 0.061°. These outcomes resulted because the negative Y-axis error of the accelerometer for zero bias (Table 2) and the positively increasing Y-axis accelerometer bias cancel each other, thereby reducing the roll error. As shown in Figure 7, as the magnetometer bias increased from 0 to 0.5 mG, the yaw error increased by 0.095°, whereas the roll and pitch errors did not change. These results occurred because the Jacobian matrix elements of the roll and pitch errors with respect to the magnetometer error in Equation (23) were determined to be zero (because *a_x_* was zero in the sensor-aligned simulation).

In Figure 6 and Figure 7, the Euler angle errors increase linearly as the sensor bias increases because the Euler angle errors are described as a linear equation with respect to the sensor error factors, as in Equation (26). Among these, the scale and installation errors are deterministic errors that occur during sensor manufacturing, whereas the offset errors vary with time and temperature. Therefore, once the deterministic errors are determined to be constant, Equation (26) can be rewritten as a linear equation with respect to the offset error as follows:

(28) $$\left[\begin{array}{c} \delta \phi \\ \delta \theta \\ \delta \psi \end{array}\right]=W_{3\times 6}\left[\begin{array}{c} o_{A}\\ o_{M} \end{array}\right]+v_{3\times 1},$$

where **W** is a matrix for converting the offset error to the Euler angle error derived from linear transformation matrix **T,** and **v** is the Euler angle error vector induced by the scale and installation errors based on Equation (26).

**Figure 8:**
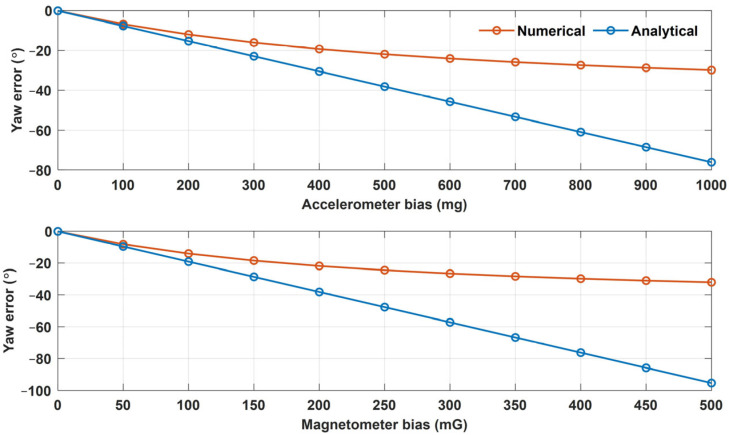
The yaw error results from the numerical and analytical approaches according to the accelerometer and magnetometer biases (unit: °).

However, Equation (26) is a linearized equation only for small error factors, whereas the actual relationship between the Euler angle error and the offset error is nonlinear. As an example, Figure 8 shows the yaw error results of the analytical and numerical approaches as the accelerometer bias increased from 0 to 1000 mg and the magnetometer bias increased from 0 to 500 mG. These results indicate that the actual error is nonlinear with respect to bias. However, the accelerometer and magnetometer biases of commercial IMUs are less than 10 mg and 5 mG, respectively, and the Euler angle errors can be approximated using a linear equation. For example, when the accelerometer bias was 10 mg, the roll errors from the numerical and analytical approaches were −0.4711° and −0.4762°, respectively; the pitch errors were both −0.6599°; and the yaw errors were −0.8690° and −0.8770°, demonstrating a difference of less than 0.01° between the two approaches. When the magnetometer bias was 5 mG, the roll errors from the numerical and analytical approaches were −0.0967° and −0.0968°, respectively; the pitch errors were −0.0946° and −0.0969°; and the yaw errors were −1.0525° and −1.0688°, demonstrating a difference of less than 0.016° between the two approaches. Given the insignificant differences between the two approaches, it is reasonable to use a linearized error model for small errors.

**Figure 9:**
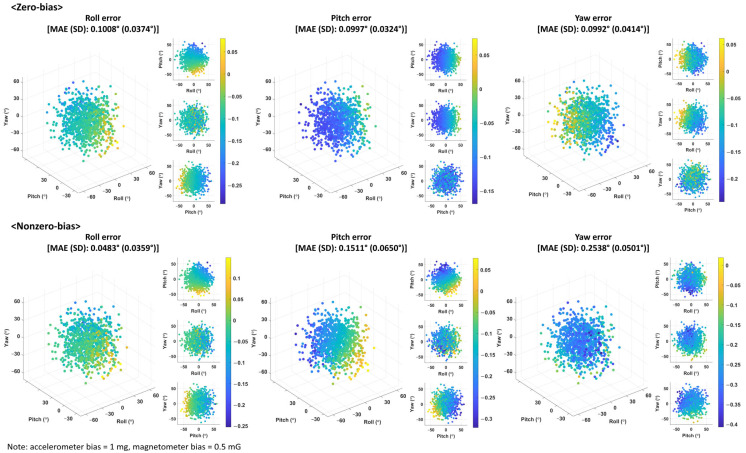
Scatter plot and mean absolute error (MAE) results with standard deviation (SD) of Euler angle error obtained via analytical formulation for zero-bias and nonzero-bias offset errors according to sensor orientation (Euler angles) in sensor-misaligned simulation of 1000 trials (unit: °).

The following are the sensor-misaligned simulation results of 1000 trials, in which arbitrary sensor orientation was simulated for each trial. In this simulation, the Euler angle errors for a zero-bias offset error and a nonzero-bias offset error (1 mg for the accelerometer and 0.5 mG for the magnetometer) were compared. Figure 9 shows 2D and 3D scatter plots of the average Euler angle errors from the analytical formulation, according to the sensor orientation (Euler angles), and the mean absolute error and standard deviation of the average Euler angle errors for 1000 simulation trials. These results indicate that the Euler angle error changes depending on sensor orientation. Because Jacobian matrix **J** and conversion matrix **C** in Equation (27) are composed of sensor signals that depend on sensor orientation, as in Equation (5), the variation in sensor orientation leads to changes in matrices **J** and **C**. The trends for cases with strong correlations between the Euler angles and their errors in Figure 8 are as follows. In the zero-bias results, the roll error decreases as the pitch angle of the sensor increases, whereas the pitch and yaw errors increase and decrease as the roll angle of the sensor increases, respectively. In the nonzero-bias results, both the roll and pitch errors decreased as the pitch angle of the sensor increased, whereas the yaw error decreased as the yaw angle of the sensor increased. These findings suggest that the relationship between sensor orientation and the Euler angle errors depends on the presence and magnitude of constant bias. The standard deviations between the simulations are larger for the nonzero-bias results than for the zero-bias results. For example, the standard deviation of the pitch errors was 0.0324° for zero bias and 0.0650° for nonzero bias, representing an increase of 0.0326°.

**Figure 10:**
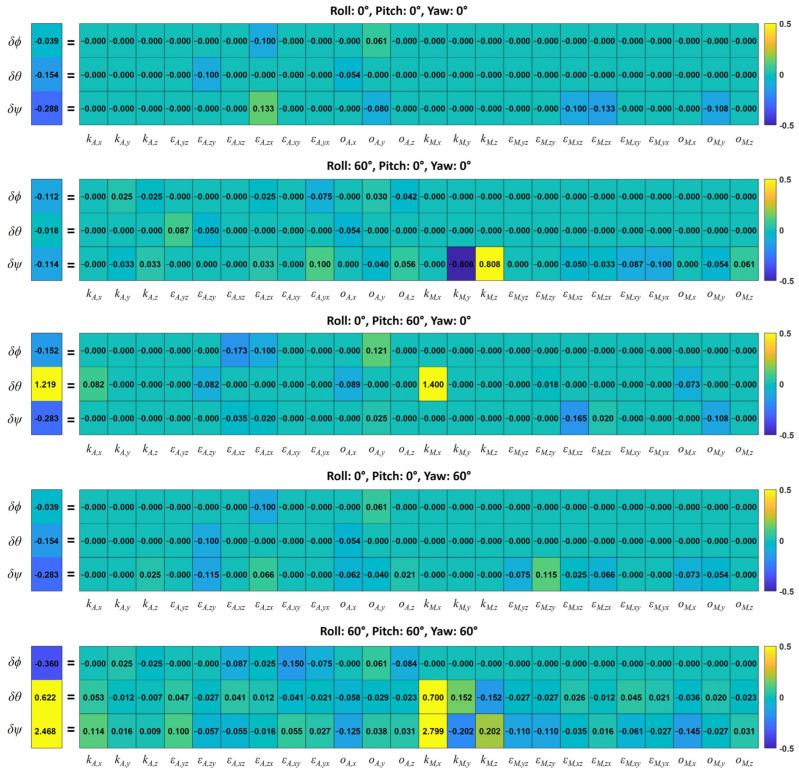
Total Euler angle errors and partial errors induced by each sensor error factor from analytical formulation for five sensor orientations (unit: °).

In the analytical formulation, the Euler angle errors are described as the sum of the errors induced by each sensor error factor according to Equation (26). Thus, the Euler angle errors for each error factor can be analyzed. Figure 10 shows the total Euler angle errors and partial errors induced by each sensor error factor for the five sensor orientations. These results show the extent to which each error factor affects the Euler angle error and how the effects change depending on sensor orientation. Among the accelerometer error factors, their contributions to the Euler angle errors were the greatest in the order of the installation error, the offset error, and the scale error. However, the maximum error remained within 0.18°. Among the magnetometer error factors, the scale error was found to have the greatest impact on the Euler angle errors. For example, the X-axis scale factor of the magnetometer did not affect the Euler angle errors at all when the pitch was 0°, but it caused errors when the pitch deviated from 0°. In particular, it caused the largest proportion of yaw errors (2.799°) when the roll, pitch, and yaw were 60°.

Table 4 presents the partial Euler angle errors induced by the accelerometer and magnetometer errors. The accelerometer errors affected all the three angles (roll, pitch, and yaw), whereas the magnetometer errors influenced only the yaw error when the pitch was 0° and both the pitch and yaw errors when the pitch was not 0°. Both the pitch (only when the pitch was not 0°) and yaw errors were more significantly influenced by the magnetometer errors than by the accelerometer errors. For example, when all the Euler angles were 60°, the pitch and yaw errors caused by the accelerometer errors were −0.0642° and 0.1368°, respectively, whereas those caused by the magnetometer errors were 0.6862° and 2.3310°, respectively.

The numerical and analytical approaches were compared for the sensor-aligned simulations. Because the numerical approach relies on numerical calculations and does not require complex formulations, it is more accurate and efficient. However, analytical formulations involve simplification and linearization, which can cause inaccuracies and result in complex formulas. Nevertheless, the presented analytical formulation can help analytically interpret the causes of the Euler angle errors using the Jacobian and conversion matrices. In addition, as discussed above, the analytical formulation is meaningful because it can analyze the Euler angle errors induced by each sensor error factor.

In [40], analytical error formulations were developed for four alignment methods, namely, the TRIAD, quaternion estimator (QUEST), factored quaternion algorithm (FAQ), and arctangent (ATAN) methods, based on the assumption that the sensor frame is perfectly aligned with the navigation frame. Therefore, the error formulations are simply composed of the magnitudes of gravitational acceleration; Earth’s magnetic field; magnetic declination and dip angles; and sensor errors. In contrast, the formulation presented in this study is applicable, regardless of the alignment between the sensor and navigation frames; however, it is composed of a more complex form than that presented in [40]. Furthermore, it is difficult to formulate the orientation errors as linear equations for alignment methods involving complex operations. Therefore, this study selected TRIAD as a representative stationary alignment method owing to its simplicity and computational efficiency. In contrast, QUEST, for example, involves a process such as calculating eigenvalues [40], which makes it difficult to derive a linear equation, such as the presented formulation. Therefore, to apply the proposed method to other stationary alignment methods, it is necessary to confirm the possibility of deriving an error formulation using a linear equation.

## 4. Conclusions

This paper presents a formulation that analytically describes the linear relationship between sensor and Euler angle errors in stationary alignments based on accelerometer and magnetometer signals. The Euler angle errors from the presented formulation were investigated for sensor-aligned and sensor-misaligned simulations, as well as compared with the errors obtained via a numerical approach. In the comparison results for the sensor-aligned simulation, the difference between the two approaches was within 0.001°, indicating that the inaccuracy of the analytical error formulations owing to linearization was negligible. The sensor-misaligned simulation results showed that the presented formulation can analyze the total Euler angle errors, as well as the partial Euler angle errors induced by the error factors of the accelerometer and the magnetometer for various sensor orientations. Although an analytical formulation is not necessarily required to obtain the Euler angle errors through a numerical approach, the presented formulations are essential for analytically investigating the effects of each sensor error factor on the Euler angle errors.

The main contributions of this study are twofold.

(i) The analytical formulation for arbitrary stationary alignment conditions: Unlike the previous studies that analyzed errors in sensor-aligned situations in which the Euler angles were 0°, this study presents a method for analyzing the Euler angle error in sensor-misaligned situations in which the Euler angles are arbitrary. This generalization is crucial for practical applications because the errors vary depending on sensor orientation. The ability to analyze and describe these errors across arbitrary alignment conditions is a key contribution that distinguishes this work.
(ii) The linear relationship between the sensor error factors and the Euler angle errors: The previous studies have primarily focused on describing the relationship between the sensor signal and Euler angle errors. In contrast, our work goes further by deriving a linear relationship between the sensor error factors (e.g., biases and misalignments) and the Euler angle errors. This distinction is meaningful because it simplifies the interpretation and analysis of the propagation of specific sensor error factors into attitude estimation errors. To the best of our knowledge, this is the first study to present an analytical formulation of the relationship between sensor and Euler angle errors for an arbitrary stationary alignment based on accelerometers and magnetometers. Although this study does not directly propose calibration or compensation methods, the presented framework serves as a foundation for future research aimed at optimizing error mitigation strategies.

The limitations of this study include (i) the lack of experimental results and (ii) the evaluation being limited to TRIAD as a stationary alignment method. Furthermore, (iii) TRIAD was performed without normalization to unitary vectors or orthogonalization. Therefore, our future studies will aim to (i) design an experimental procedure to validate the findings by testing controllable error factors, (ii) extend the formulation to other alignment methods, such as QUEST and ATAN, for the broader analysis of alignment methods, and (iii) develop a formulation for TRIAD that includes vector normalization and orthogonalization. In addition, because this study enables the analysis of Euler angle errors under arbitrary alignment conditions, it is also possible to derive an optimal Euler angle combination that can minimize the Euler angle errors for specific sensor errors. This topic is reserved for future research.

## Figures and Tables

**Table 1:** Sensor error factors of accelerometer and magnetometer.

Error Factor	Accelerometer (Axis)	Magnetometer (Axis)
Scale error	0.1% (X, Y, Z)	0.1% (X, Y, Z)
Installation error	0.1° (YZ, ZY, XZ, ZX, XY, YX)	0.1° (YZ, ZY, XZ, ZX, XY, YX)
Noise density	0.15 mg/√Hz (X, Y, Z)	0.17 mG/√Hz (X, Y, Z)
Bias instability	0.07 mg (X, Y, Z)	0.03 mG (X, Y, Z)
Constant bias	0–1 mg (X, Y, Z)	0–0.5 mG (X, Y, Z)

**Table 2:** Average error (with standard deviation) results of accelerometer and magnetometer signals in sensor-aligned simulation.

	X	Y	Z
Accelerometer [mg]	1.6916 (1.5095)	−1.6894 (1.4652)	0.8443 (1.5098)
Magnetometer [mG]	−0.4570 (1.7111)	1.2875 (1.6604)	−1.0525 (1.7097)

**Table 3:** Average error (with standard deviation) results of Euler angle errors from numerical and analytical approaches in sensor-aligned simulation [unit: °].

	X	Y	Z
Numerical approach	−0.0967 (0.0839)	−0.0962 (0.0856)	−0.1163 (0.4277)
Analytical approach	−0.0968 (0.0840)	−0.0969 (0.0865)	−0.1167 (0.4276)
Mean absolute difference (×10^−3^)	0.1476	0.8107	2.4411

**Table 4:** Partial errors induced by accelerometer and magnetometer errors from analytical formulations for five sensor orientations [unit: °].

Euler Angle (Roll/Pitch/Yaw)	Roll Error		Pitch Error		Yaw Error	
	Acc	Mag	Acc	Mag	Acc	Mag
0/0/0	−0.0395	0.0000	−0.1542	0.0000	0.0524	−0.3404
60/0/0	−0.1118	0.0000	−0.0176	0.0000	0.1484	−0.2620
0/60/0	−0.1522	0.0000	−0.0900	1.3094	−0.0308	−0.2523
0/0/60	−0.0395	0.0000	−0.1542	0.0000	−0.1052	−0.1778
60/60/60	−0.3602	0.0000	−0.0642	0.6862	0.1368	2.3310

Note: Acc = accelerometer; Mag = magnetometer.

## Data Availability

The original contributions presented in this study are included in the article. Further inquiries can be directed to the corresponding author.
